# Advances in adoptive cellular therapy of cancer

**DOI:** 10.1186/1479-5876-13-S1-K16

**Published:** 2015-01-15

**Authors:** David Stroncek

**Affiliations:** 1 Department of Transfusion Medicine, Clinical Center, National Institutes of Health, Bethesda, Maryland USA

## 

Adoptive cellular therapy is becoming an important cancer therapy. Tumor infiltrating T cells and peripheral blood T cells engineered to express high affinity T cell receptors specific to tumor antigens have been effective in treating melanoma. T cells engineered to express chimeric antigen receptors are being used to treat cancers and hematologic malignancies. Clinical studies have found that the characteristics of cellular therapies effects clinical outcomes. There is considerable variability between production lots of adoptive cellular therapies. This variability affects product characteristics and clinical outcomes. Production variability is due to both donor and manufacturing factors. Donor genetics as well as donor age, gender, race and prior therapies may affect product characteristics and function. The complexity involved with manufacturing cellular therapies also contributes to variability in the final product. Manufacturing adoptive cell therapies may involve stimulation with antibodies, cytokines, growth factors or allogeneic cells; prolonged culture and expansion; and genetic engineering. Product characteristics can affect clinical outcomes. Several studies have found that adoptive cellular therapies that have greater *in vivo* persistence and proliferation are associated with better clinical outcomes; however, the specific product characteristics responsible for better or worse clinical outcomes are not well understood. To better understand the critical characteristics responsible for the clinical effectiveness of cellular therapies we and others have been studying the potency of cellular therapies. Specific phenotypes have been associated with better clinical outcomes. Animal models suggest that T memory stem cells have greater proliferative potential and persistence than effector T cells and are more effective for adoptive cellular therapy. We have found that high throughput molecular assays are useful at identifying markers that are useful at measuring cell potency. In conclusion, in order to improve the clinical effectiveness of cellular therapies it’s important to better understand the biological functions that contribute to product potency and develop potency markers and assays to ensure that the highest quality cells are consistently produced.

